# IL-4 and IL-13 Promote Proliferation of Mammary Epithelial Cells through STAT6 and IRS-1

**DOI:** 10.3390/ijms222112008

**Published:** 2021-11-05

**Authors:** Wan-Ju Wu, Sue-Hong Wang, Chun-Chi Wu, Yi-An Su, Chin-Yin Chiang, Ching-Hong Lai, Tsung-Hsiang Wang, Tsung-Lin Cheng, Jia-Yu Kuo, Tsai-Ching Hsu, Ting-Hui Lin, Yi-Ju Lee

**Affiliations:** 1 Department of Obstetrics and Gynecology, Changhua Christian Hospital, Changhua 500, Taiwan; crystalwu835@gmail.com; 2 Department of Biomedical Sciences, Chung Shan Medical University, Taichung 402, Taiwan; wangsh@csmu.edu.tw; 3 Institute of Medicine, Chung Shan Medical University, Taichung 402, Taiwan; daniel@csmu.edu.tw (C.-C.W.); nancy.yih.an.su@gmail.com (Y.-A.S.); kevin503able@gmail.com (C.-Y.C.); 090823@nhri.edu.tw (C.-H.L.); sean860310@gmail.com (T.-H.W.); lyn5200258@gmail.com (T.-L.C.); xxc55663@gmail.com (J.-Y.K.); htc@csmu.edu.tw (T.-C.H.); 4 Department of Health Diet and Industry Management, Chung Shan Medical University, Taichung 402, Taiwan; 5 Immunology Research Center, Chung Shan Medical University, Taichung 402, Taiwan; 6 Department of Medical Research, Chung Shan Medical University Hospital, Taichung 402, Taiwan

**Keywords:** IL-4, IL-13, mammary glands, cell proliferation, STAT6, IRS protein

## Abstract

T helper (Th)2 cytokines such as interleukin (IL)-4 and IL-13 control immune function by acting on leukocytes. They also regulate multiple responses in non-hematopoietic cells. During pregnancy, IL-4 and IL-13 facilitate alveologenesis of mammary glands. This particular morphogenesis generates alveoli from existing ducts and requires substantial cell proliferation. Using 3D cultures of primary mouse mammary epithelial cells, we demonstrate that IL-4 and IL-13 promote cell proliferation, leading to enlargement of mammary acini with partially filled lumens. The mitogenic effects of IL-4 and IL-13 are mediated by STAT6 as inhibition of STAT6 suppresses cell proliferation and improves lumen formation. In addition, IL-4 and IL-13 stimulate tyrosine phosphorylation of insulin receptor substrate-1 (IRS-1). Prolonged treatment with these cytokines leads to increased IRS-1 abundance, which, in turn, amplifies IL-4- and IL-13-stimulated IRS-1 tyrosine phosphorylation. Through signaling crosstalk between IL-4/IL-13 and insulin, a hormone routinely included in mammary cultures, IRS-1 tyrosine phosphorylation is further enhanced. Lowering IRS-1 expression reduces cell proliferation, suggesting that IRS-1 is involved in IL-4- and IL-13-stimulated cell proliferation. Thus, a Th2-dominant cytokine milieu during pregnancy confers mammary gland development by promoting cell proliferation.

## 1. Introduction

Interleukin (IL)-4 and IL-13 are secreted by T helper (Th)2 cells, basophils, eosinophils, mast cells, and group 2 innate lymphoid cells (ILC2). These cytokines contribute to the protective and pathogenic features of type II immunity, such as protecting against helminth infection and eliciting allergic responses, respectively [1,2]. IL-4 stimulates proliferation and isotype switching to IgE in B cells, as well as promotes differentiation of Th cells into the Th2 lineage by induction of GATA-binding protein 3 (GATA3). IL-4 and IL-13 also drive macrophage polarization toward the M2 phenotype. IL-13 is a crucial cytokine for mucus secretion, smooth muscle contraction, and airway hyperresponsiveness [3].

IL-4 and IL-13 exert their functions by signaling through cell surface receptors. IL-4 binds to IL-4Rα, which dimerizes with common γ chain (γ_c_ chain) to form the type I receptor or with IL-13Rα1 to form the type II receptor. IL-13 activates the type II receptor since it binds to IL-13Rα1. IL-13 also binds to IL-13Rα2 with much higher affinity. IL-4Rα and IL-13Rα1 are ubiquitously expressed, while the expression of γ_c_ chain is restricted to hematopoietic cells. IL-13Rα2 is found in epithelial cells and fibroblasts. For the type I and type II receptors, ligand binding triggers activation of JAK kinases, resulting in tyrosine phosphorylation of STAT6 and insulin receptor substrate (IRS)-1/IRS-2 [4,5]. IL-13Rα2 is considered as a decoy receptor for IL-4/IL-13 signaling, but recent evidence has revealed its role in activating the ERK/AP-1 and the Src/PI3K/AKT/mTOR pathways [6,7].

Development of mammary glands occurs primarily after birth. During pregnancy, mammary cells proliferate and bud from existing ducts to form alveoli. Abundant alveolar cells synthesize and secrete milk during lactation. Mammary alveolus displays spherical morphology, with bi-layered epithelial cells composed of luminal epithelial cells and myoepithelial cells, and a central lumen. The epithelium is separated from the surrounding stroma by an intact basement membrane [8]. The structure and function of mammary alveoli can be recapitulated in vitro by culturing mammary cells on a reconstituted basement membrane matrix along with the proper hormones and growth factors [9].

There is increasing evidence that the immune system controls tissue development and organogenesis [10]. In mammary glands, Th1 cells, through interaction with epithelial-associated CD11c^+^ antigen-presenting cells, negatively regulate postnatal organogenesis via their effector cytokine, interferon-γ (IFN-γ) [11]. In contrast, IL-4 stimulates the promoter activity of the *β-casein* gene, a milk protein gene that is often used to indicate mammary differentiation [12]. Th2 cytokines (IL-4 and IL-13) also promote alveologenesis during pregnancy as ablation of both *Il-4* and *Il-13* genes or just the *Stat6* gene results in delayed alveologenesis [13,14]. GATA-3, the master regulator of Th2 polarization, has been shown to specify and maintain luminal cell fate in mammary glands [15,16]. Given the significance of Th2 cytokines in mammary gland development, we examined the influence of IL-4 and IL-13 on structure and function of mammary cells in 3D cultures and found that IL-4 and IL-13 stimulate cell proliferation via STAT6 and IRS-1.

## 2. Results

### 2.1. IL-4 and IL-13 Stimulate β-Casein Expression and Enlargement of Mammary Acini

**Figure 1:**
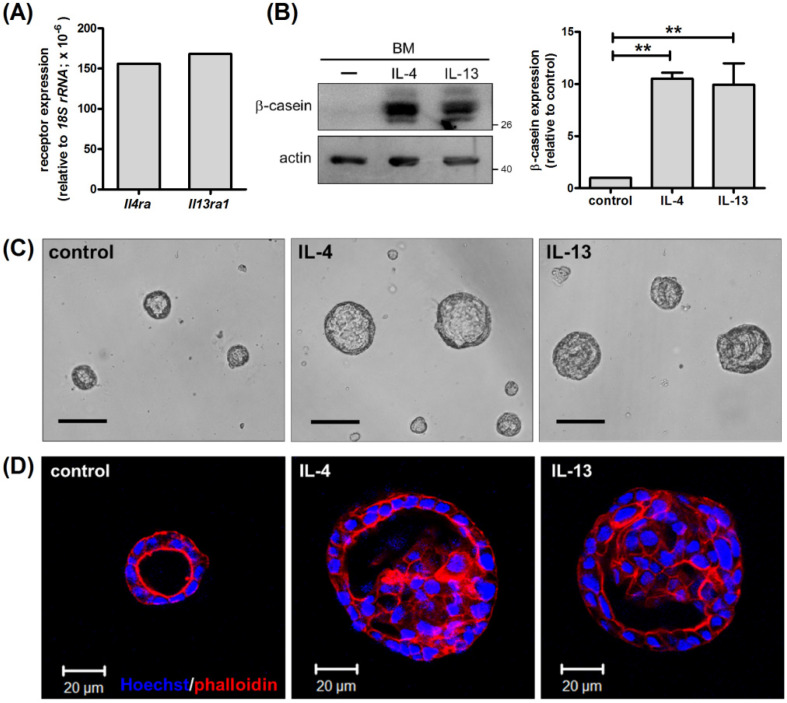
IL-4 and IL-13 stimulate β-casein expression and enlargement of mammary acini. Primary mouse mammary epithelial cells were cultured on a basement membrane (BM)-like matrix, Matrigel. (**A**) Expression of *Il4ra* and *Il13ra1* mRNA was analyzed by real-time RT-PCR. (**B**–**D**) Cells were untreated or treated with IL-4 (50 ng/mL) or IL-13 (50 ng/mL) for 2 d. (**B**) Total cell lysates were analyzed by immunoblotting with antibodies to β-casein and actin. Actin was used as a loading control. Data were quantified, normalized to loading control and expressed as fold change relative to control (*n* = 3). ** *p* < 0.01. (**C**) Photographs of untreated, IL-4-treated and IL-13-treated mammary acini. Scale bar, 100 μm. (**D**) Cells were stained with rhodamine phalloidin (red) and Hoechst 33342 (blue) and subjected to confocal microscopy. Images were taken from the center of acini. Scale bar, 20 μm. Unless otherwise noted, mammary cells were always cultured in the presence of insulin and hydrocortisone.

Prolactin stimulates β-casein expression via STAT5 in mammary cells [17]. IL-4 does so through its major signaling molecule STAT6 in the mammary epithelial cell line HC11 [12]. To confirm this and test the effects of another Th2 cytokine, IL-13, which also activates STAT6, we cultured primary mouse mammary epithelial cells on a basement membrane-like matrix, Matrigel, and stimulated them with IL-4 or IL-13. At first, we confirmed expression of IL-4 and IL-13 receptors, IL-4Rα and IL-13Rα1, in mammary cells (Figure 1A). Stimulation with IL-4 and IL-13 for 2 d caused induction of β-casein, which is consistent with previous findings (Figure 1B) [12].

Mammary cells cultured on Matrigel adopt an acinar morphology with a central lumen, resembling alveoli of mammary glands in vivo [18]. Mammary acini treated with IL-4 or IL-13 were enlarged (Figure 1C), suggesting that IL-4 and IL-13 stimulate cell proliferation. Given the large size of IL-4- and IL-13-treated acini, we wondered if lumens were properly formed. As shown in Figure 1D, untreated acini displayed a single layer of epithelial cells with a hollow lumen. The strong staining of phalloidin pointed to the localization of F-actin, particularly on the apical surfaces of acini. In response to IL-4 and IL-13, lumens were partially filled (Figure 1D). As lumen formation requires growth arrest and apoptosis of cells inside mammary spheres, luminal filling in IL-4- and IL-13-treated acini might be due to enhanced cell proliferation or survival [19].

### 2.2. IL-4 and IL-13 Stimulate Proliferation of Mammary Cells

**Figure 2:**
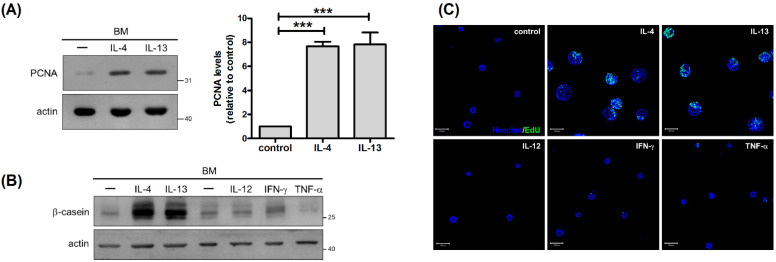
IL-4 and IL-13 promote proliferation of mammary cells. (**A**) Mammary cells cultured on a basement membrane (BM)-like matrix, Matrigel, were untreated or treated with IL-4 or IL-13 for 2 d. Total cell lysates were analyzed by immunoblotting with antibodies to PCNA and actin. Actin was used as a loading control. Data were quantified, normalized to loading control and expressed as fold change relative to control (*n* = 4). *** *p* < 0.005. (**B**,**C**) Cells were untreated or treated with IL-4, IL-13, IL-12 (20 ng/mL), IFN-γ (10 ng/mL), or TNF-α (10 ng/mL) for 2 d. (**B**) Total cell lysates were analyzed by immunoblotting with antibodies to β-casein and actin. (**C**) Prior to harvesting, cells were pulsed with EdU (10 μM; green) for 8 h. Nuclei were stained with Hoechst 33342 (blue). Scale bar, 100 μm. In these images, clusters of nuclei indicate cells within mammary acini, and IL-4 and IL-13 stimulate EdU incorporation into cells within mammary acini.

To investigate whether IL-4- and IL-13-stimulated enlargement of mammary acini can be ascribed to enhanced cell proliferation, we examined the expression of proliferating cell nuclear antigen (PCNA). IL-4 and IL-13 augmented PCNA expression, implying their positive impact on cell proliferation (Figure 2A). To further demonstrate this phenomenon, 5-ethynyl-2′-deoxyuridine (EdU) incorporation was measured to evaluate DNA synthesis. Higher amounts of EdU were incorporated into cells treated with IL-4 and IL-13, confirming the mitogenic effect of these cytokines (Figure 2C). These results indicated that IL-4 and IL-13 stimulate proliferation of mammary cells, leading to enlargement of mammary acini. To ascertain that the stimulatory effect of IL-4 and IL-13 on β-casein expression and cell proliferation is not a general event for any cytokines, we examined these responses in cells treated with IL-12, IFN-γ, and tumor necrosis factor-α (TNF-α). IL-12 promotes differentiation of naïve T cells into Th1 cells, and IFN-γ and TNF-α are Th1 cytokines. None of these cytokines stimulated β-casein expression (Figure 2B) and cell proliferation (Figure 2C), verifying the specific effect of IL-4 and IL-13 on mammary functions.

### 2.3. STAT6 Mediates the Pro-Proliferative Effect of IL-4 and IL-13

**Figure 3:**
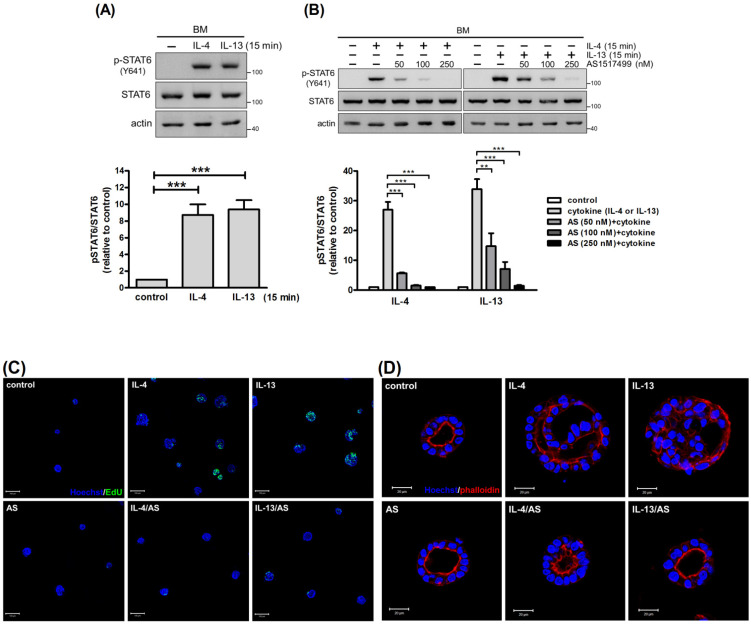
Inhibition of STAT6 alleviates the pro-proliferative effect of IL-4 and IL-13. (**A**) Mammary cells cultured on a basement membrane (BM)-like matrix, Matrigel, were untreated or treated with IL-4 or IL-13 for 15 min (*n* = 4). (**B**) Mammary cells cultured on Matrigel were pretreated with STAT6 inhibitor AS1517499 (50~250 nM) for 1 h and stimulated with IL-4 or IL-13 for 15 min (*n* = 3). Total cell lysates were analyzed by immunoblotting with antibodies to phospho-STAT6 (p-STAT6), STAT6 and actin. Actin was used as a loading control. Data were quantified, normalized to loading control and expressed as fold change (pSTAT6/STAT6) relative to control. ** *p* < 0.01, *** *p* < 0.005. (**C**,**D**) Mammary cells cultured on Matrigel were pretreated with AS1517499 (100 nM) for 1 h and stimulated with IL-4 or IL-13 for 1 d. (**C**) Prior to harvesting, cells were pulsed with EdU (green) for 8 h. Nuclei were stained with Hoechst 33342 (blue). Scale bar, 100 μm. (**D**) Cells were stained with rhodamine phalloidin (red) and Hoechst 33342 (blue), and subjected to confocal microscopy. Images were taken from the center of acini. Scale bar, 20 μm.

To unravel the underlying signaling mechanism of IL-4- and IL-13-stimulated cell proliferation, we examined the involvement of STAT6, the major signaling molecule in IL-4/IL-13 signaling pathway. Exposure to IL-4 or IL-13 for 15 min resulted in tyrosine phosphorylation of STAT6 (Figure 3A), confirming STAT6 activation in mammary cells. STAT6 inhibitor AS1517499 suppressed IL-4- and IL-13-induced tyrosine phosphorylation of STAT6 in a dose-dependent manner (Figure 3B). At a concentration of 100 nM, AS1517499 decreased IL-4- and IL-13-induced expression of *Ym1*, a target gene of STAT6, without eliciting cytotoxic effect (Figure S1A,B) [20]. Although optimal cell growth was observed following cytokine treatment for 2 d (Figure 2C), STAT6 inhibitor seemed to lose its activity during this period. Much higher concentrations of AS1517499 were required to inhibit IL-4- and IL-13-stimulated phosphorylation of STAT6 (Figure S1C). Thus, a shorter treatment course (1 d) was employed to examine the effect of AS1517499. Under this condition, IL-4 and IL-13 stimulated incorporation of EdU into cells. AS1517499 reduced IL-4- and IL-13-stimulated proliferation, prevented acinus enlargement and improved lumen formation (Figure 3C,D). These results demonstrated that IL-4 and IL-13 stimulate proliferation of mammary cells through STAT6.

### 2.4. IL-4 and IL-13 Stimulate Tyrosine Phosphorylation and Expression of IRS-1

**Figure 4:**
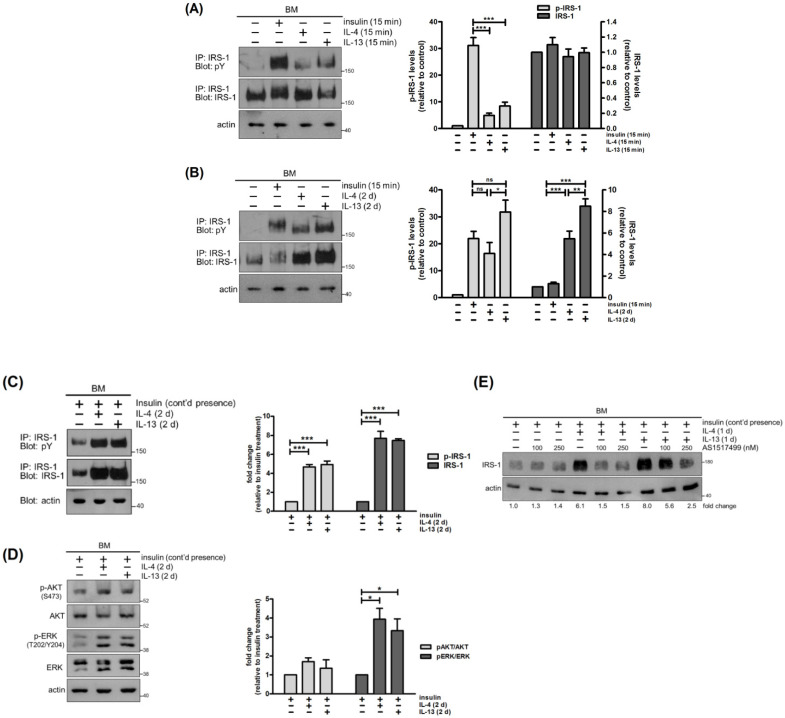
IL-4 and IL-13 stimulate tyrosine phosphorylation and expression of IRS-1. Mammary cells were cultured on a basement membrane (BM)-like matrix, Matrigel, either in the absence of insulin (**A**,**B**) or in the continued presence of insulin (**C**–**E**). (**A**) Cells were untreated or stimulated with insulin (100 nM), IL-4 or IL-13 for 15 min (*n* = 5). (**B**) Cells were untreated for 2 d, untreated for 2 d and then stimulated with insulin for 15 min, or treated with IL-4 or IL-13 for 2 d (*n* = 5). (**C**) Cells were stimulated with IL-4 or IL-13 for 2 d in the continued presence of insulin (*n* = 3). Total cell lysates were immuoprecipitated (IP) with antibody to IRS-1 followed by immunoblotting with antibody to phosphotyrosine (pY). Blots were stripped and reprobed with antibody to IRS-1. Total cell lysates were also analyzed by immunoblotting with antibody to actin. Actin was used as a loading control. (**D**) Cells were stimulated with IL-4 or IL-13 for 2 d in the continued presence of insulin. Total cell lysates were analyzed by immunoblotting with antibodies to phospho-AKT (p-AKT), AKT, phospho-ERK (p-ERK), ERK and actin. (*n* = 3) (**E**) Cells were pretreated with STAT6 inhibitor AS1517499 (100 nM, 250 nM) for 1 h and stimulated with IL-4 or IL-13 for 1 d in the continued presence of insulin. Total cell lysates were analyzed by immunoblotting with antibodies to IRS-1 and actin. Data were quantified, normalized to loading control and expressed as fold change relative to control (**A**,**B**,**E**) or insulin treatment (**C**,**D**). * *p* < 0.05, ** *p* < 0.01, *** *p* < 0.005; and ns, not significant.

In addition to STAT6, IRS-1 is another key molecule in IL-4 and IL-13 signaling [21]. It is also involved in insulin and insulin-like growth factor (IGF) signal transduction [22]. Given the essential role of IRS-1 in IL-4-stimulated mitogenesis in hematopoietic cells, it is of interest to find out whether IRS-1 also contributes to proliferation of mammary cells [23]. Firstly, we examined IRS-1 tyrosine phosphorylation in response to IL-4 and IL-13. Using insulin as a positive control, we found that treatment with IL-4 and IL-13 for 15 min caused tyrosine phosphorylation of IRS-1. However, the effect was less pronounced than that induced by insulin (Figure 4A). Prolonged cytokine treatment led to an increase in levels of IRS-1 tyrosine phosphorylation and expression (Figure 4B). The ratios of IRS-1 phosphorylation to IRS-1 expression (p-IRS-1/IRS-1) were comparable among cells subjected to short and prolonged exposure to cytokines (Figure S2A,B), suggesting that prolonged cytokine treatment augments IRS-1 tyrosine phosphorylation by enhancing its protein abundance. In other words, this treatment does not upregulate JAK activity, but substantially increases amounts of JAK substrate, IRS-1, to be phosphorylated (Figure S2C).

After 2 days of incubation, IRS-1 levels were reduced in untreated cells (Figure 4B). It is likely that IRS-1 was degraded due to serum starvation, but inhibition of proteasome by MG132 did not restore IRS-1 levels (data not shown). IL-4 and IL-13 elevated IRS-1 protein abundance (Figure 4B). This is, at least in part, controlled at the transcriptional level since *Irs1* mRNA levels were enhanced by 2-fold (Table S1). However, a 5- to 8-fold increase in IRS-1 protein abundance was observed (Figure 4B), suggesting that post-transcriptional mechanisms such as regulation of protein stability might be involved.

Mammary cells were routinely cultured with insulin (Materials and Methods). In Figure 4A,B, insulin was omitted from cultures in order to examine IRS-1 tyrosine phosphorylation induced by IL-4 or IL-13 alone. We then assessed IRS-1 tyrosine phosphorylation under continued presence of insulin and prolonged treatment with cytokines, a condition used for evaluating β-casein expression and cell proliferation. IRS-1 tyrosine phosphorylation was readily detected in cells cultured with insulin. Application of IL-4 and IL-13 further increased levels of IRS-1 phosphorylation and expression (Figure 4C). Of note, lower amounts of samples were used in these experiments, due to their strong signals. Tyrosine phosphorylated IRS recruited signaling molecules such as PI3K and Grb2, resulting in activation of AKT and ERK [24,25]. Combined treatment with insulin and IL-4/IL-13 augmented ERK phosphorylation with marginal influences on AKT activation, despite an increase in association of PI3K with IRS-1 (Figure 4D and data not shown).

We next examined the signaling mechanism for upregulation of IRS-1, focusing on the role of STAT6, which is strongly and rapidly activated by IL-4 and IL-13 (Figure 3A). Due to the fact that STAT6 inhibitor AS1517499 does not last for 2 d, cells were subjected to treatment for 1 d. AS1517499 alleviated the stimulatory effect of IL-4 and IL-13 on IRS-1 expression without altering basal levels of IRS-1. It was more effective against the effects of IL-4 than those of IL-13 (Figure 4E), which is consistent with the impact of AS1517499 on STAT6 phosphorylation (Figure 3B). Taken together, IL-4 and IL-13 stimulate not only tyrosine phosphorylation, but also expression of IRS-1, in mammary cells. IRS-1 that is upregulated after prolonged cytokine treatment can be phosphorylated by cytokine receptor-associated JAKs and insulin receptor kinase, leading to signal amplification.

**Figure 5:**
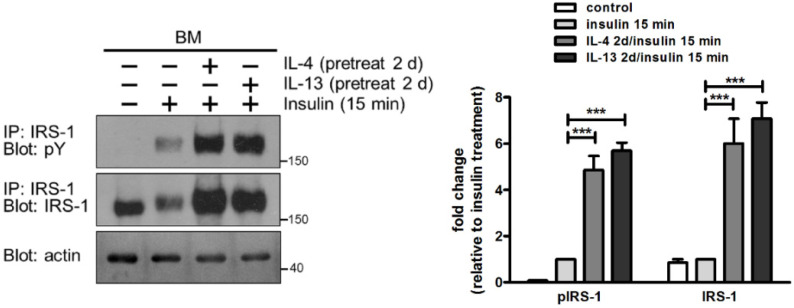
Pretreatment with IL-4 and IL-13 boosts insulin-stimulated tyrosine phosphorylation of IRS-1. Mammary cells were cultured on a basement membrane (BM)-like matrix, Matrigel, in the absence of insulin. Cells were pretreated with IL-4 or IL-13 for 2 d and then stimulated with insulin for 15 min. Total cell lysates were immunoprecipitated (IP) with antibody to IRS-1, followed by immunoblotting with antibody to phosphotyrosine (pY). Blots were stripped and reprobed with antibody to IRS-1. Total cell lysates were analyzed by immunoblotting with antibody to actin. Actin was used as a loading control. Data were quantified, normalized to loading control and expressed as fold change relative to insulin treatment alone (*n* = 4). *** *p* < 0.005.

Given the key role of IRS-1 in insulin signaling, we also examined whether increased abundance of IRS-1 following pretreatment with IL-4 and IL-13 exert a priming effect to augment insulin signaling. Cells were cultured in the absence of insulin, pretreated with IL-4 or IL-13 for 2 d, and stimulated with insulin for 15 min. Pretreatment with cytokines boosted insulin signaling with enhanced IRS-1 tyrosine phosphorylation, due to elevated expression of IRS-1 (Figure 5). Collectively, we demonstrated a crosstalk between IL-4/IL-13 and insulin signaling in mammary cells. IL-4 and IL-13 weakly activate IRS-1 tyrosine phosphorylation but increase IRS-1 protein abundance, whereas insulin strongly induces IRS-1 tyrosine phosphorylation. Combined treatment with IL-4/IL-13 and insulin leads to high levels of IRS-1 tyrosine phosphorylation.

### 2.5. IRS-1 Is Involved in IL-4- and IL-13-Stimulated Cell Proliferation

**Figure 6:**
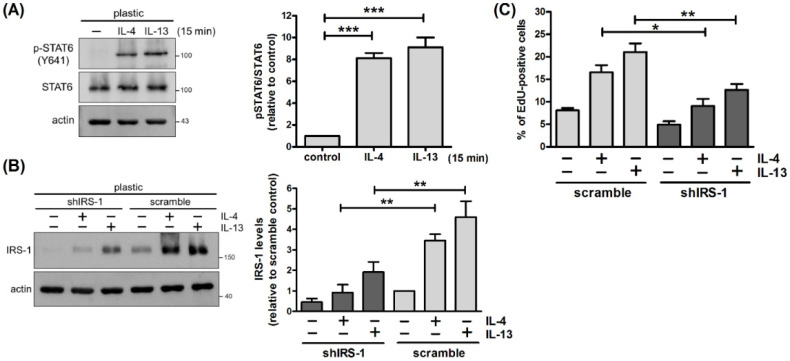
Lowering IRS-1 levels reduces IL-4- and IL-13-stimulated cell proliferation. (**A**) Mammary cells cultured on tissue culture plastic were untreated or treated with IL-4 or IL-13 for 15 min. Total cell lysates were analyzed by immunoblotting with antibodies to phospho-STAT6 (p-STAT6), STAT6 and actin. Actin was used as a loading control. Data were quantified, normalized to loading control, and expressed as fold change (pSTAT6/STAT6) relative to control (*n* = 4). *** *p* < 0.005. (**B**,**C**) Mammary cells cultured on plastic were infected with lentivirus carrying shRNA against *Irs1* (shIRS-1) or scramble RNA for 2 d, and then stimulated with IL-4 or IL-13 for 2 d. (**B**) Total cell lysates were analyzed by immunoblotting with antibodies to IRS-1 and actin. Data were quantified, normalized to loading control, and expressed as fold change relative to scramble control (*n* = 4). ** *p* < 0.01. (**C**) Prior to harvesting, cells were pulsed with EdU (10 μM) for 15 h. Nuclei were stained with Hoechst 33342. The percentage of EdU-positive cells was determined by TissueFAXS^TM^ cytometry (*n* = 4). * *p* < 0.05, ** *p* < 0.01.

**Figure 7:**
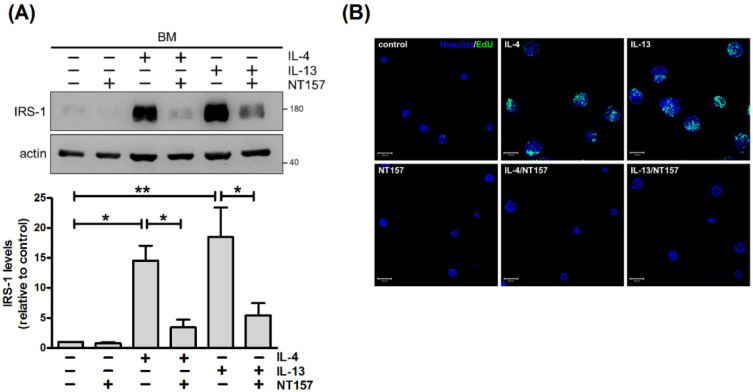
IRS-1 inhibitor NT157 suppresses IL-4- and IL-13-stimulated cell proliferation. Mammary cells cultured on a basement membrane (BM)-like matrix, Matrigel, were untreated or treated with NT157 (5 μM), IL-4 or IL-13 for 2 d. (**A**) Total cell lysates were analyzed by immunoblotting with antibodies to IRS-1 and actin. Actin was used as a loading control. Data were quantified, normalized to loading control and expressed as fold change relative to control (*n* = 4). * *p* < 0.05, ** *p* < 0.01. (**B**) Prior to harvesting, cells were pulsed with EdU (10 μM; green) for 8 h. Nuclei were stained with Hoechst 33342 (blue). Scale bar, 100 μm.

Signaling through IRS-1 elicits a number of responses, including proliferation, survival and metabolism [24,25]. We next examined whether IRS-1 plays a role in IL-4- and IL-13-stimulated cell proliferation. IRS-1 expression was reduced by RNA interference to assess the effect on proliferation. For ease of handling, mammary cells were cultured on tissue culture plastic. Unlike prolactin for which signaling requires cell adhesion on basement membrane, IL-4 and IL-13 stimulated tyrosine phosphorylation of STAT6 in cells cultured on plastic (Figure 6A) [17]. They also promoted IRS-1 expression and cell proliferation (Figure 6B,C). Lowering IRS-1 levels by infecting cells with lentivirus carrying shRNA against *Irs1* (shIRS-1) reduced IL-4- and IL-13-stimulated cell proliferation (Figure 6C). To confirm these results, IRS inhibitor NT157 which causes serine phosphorylation and subsequent degradation of IRS proteins was used [26]. NT157 diminished basal and IL-4- and IL-13-induced expression of IRS-1 (Figure 7A). It also abrogated cytokine-stimulated cell proliferation (Figure 7B). These results suggest that IL-4 and IL-13 increase IRS-1 expression, which facilitates cell proliferation.

## 3. Discussion

**Figure 8:**
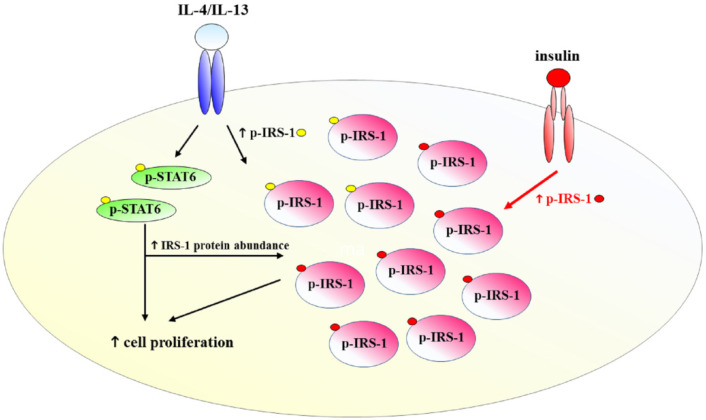
Summary diagram. A crosstalk between IL-4/IL-13 and insulin signaling occurs in mammary cells. IL-4 and IL-13 weakly activate IRS-1 tyrosine phosphorylation but increase IRS-1 protein abundance via STAT6. In the presence of insulin that is routinely included in cultures, levels of IRS-1 tyrosine phosphorylation are further enhanced. Therefore, combined treatment with IL-4/IL-13 and insulin leads to high levels of IRS-1 tyrosine phosphorylation. Both STAT6 and IRS-1 are involved in IL-4- and IL-13-stimulated cell proliferation. Small yellow circles and red circles indicate cytokine- and insulin-stimulated tyrosine phosphorylation, respectively.

Mammary gland development is controlled by various hormones and growth factors. Emerging regulators include immune mediators, such as Th2 cytokines. In this study, we demonstrated that IL-4 and IL-13 stimulate proliferation of mammary cells via STAT6 and IRS-1. This is consistent with the findings that these cytokines promote alveologenesis of mammary glands during pregnancy, a type of morphogenesis requiring extensive cell proliferation [13]. We also found that IL-4 and IL-13 not only activate IRS-1 tyrosine phosphorylation but also increase its protein abundance, leading to signal amplification. Under the routine culture condition with the presence of insulin, IRS-1 tyrosine phosphorylation is further upregulated by combined action of IL-4/IL-13 and insulin (Figure 8).

In addition to the influence of Th2 cytokines on alveologenesis, other immune cells and mediators contribute to mammary gland development [27]. Regarding the innate immune system, macrophages, eosinophils, and mast cells help ductal outgrowth and branching morphogenesis during puberty [28,29]. Mast cells and macrophages are involved in alveolar apoptosis and clearance of dead cells during involution, respectively. Both cell types also facilitate tissue remodeling in the later stage of involution [30,31]. Adaptive immunity also plays a role in mammary gland development. The most well-known example is secretion of immunoglobulin A by infiltrating plasma cells during lactation, which provides passive immunity to the newborn. In contrast to Th2 cytokines that promote development, Th1 cells inhibit postnatal organogenesis via IFN-γ [11]. Interestingly, there is increasing evidence that various leukocytes are localized in the stroma and in close contact with the epithelium in normal mammary glands, suggesting their participation in mammary gland development [32,33,34].

In this study, we demonstrated that STAT6 and IRS-1 are involved in IL-4- and IL-13-stimulated proliferation of mammary cells. The mechanistic basis of STAT6-stimulated proliferation has recently been elucidated in pancreatic cancer. In response to IL-4 and IL-13, STAT6 directly induces the expression of *Myc*, which then activates transcription of glycolytic genes. This leads to metabolic reprogramming with enhanced glycolysis [35]. IRS-1 also governs cell proliferation and metabolism. Overexpression of IRS-1 in mammary glands causes mammary hyperplasia and tumorigenesis. Through interaction with β-catenin, IRS-1 increases expression of β-catenin-regulated targets, cyclin D1 and c-myc [36]. In addition to the direct action of IRS-1 on induction of genes involved in cell cycle progression, signaling events downstream of IRS-1, such as the Ras/MAPK and PI3K/AKT/mTOR pathways, regulates cell proliferation and metabolism [24,25,37]. It has been shown that IL-4, via IL-4Rα, stimulates glutamine metabolism to support cell growth in breast cancer cells [38]. Given the role of IRS-1 in proliferation and metabolism, we speculate that upregulation of IRS-1 following IL-4/IL-13 treatment reprograms cellular metabolism to facilitate proliferation of mammary cells.

The pro-proliferative effect of IL-4 and IL-13 on other cell types has also been reported. In bronchial epithelial cells, IL-13 promotes cell proliferation by releasing transforming growth factor-α, which elicits paracrine/autocrine actions. This might confer epithelial hypertrophy in allergic asthma [39]. IL-13 also stimulates cell cycle activity in neonatal myocardiocytes and heart regeneration, possibly via ERK and AKT pathways [40]. In vascular smooth muscle cells, induction of ornithine decarboxylase by the PI3K and ERK pathways is responsible for IL-4- and IL-13-stimulated cell proliferation [41]. IL-4 and IL-13 contribute to cancer progression in various cancer types, and several mechanisms have been documented [42,43,44]. In response to these cytokines, enhanced glycolysis and glutamine metabolism support growth of pancreatic and breast cancer cells, respectively [35,38]. Upregulation of IL-13Rα2 has been detected in cancer cells, which facilitates tumor growth and metastasis. Signaling downstream of IL-13Rα2, including the ERK/AP-1, the Src/PI3K/AKT/mTOR and the PTP1B/Src/AKT and ERK pathways have been shown to mediate these effects [6,7,45]. In breast cancer cells, IL-4 exerts a wide spectrum of pro-tumorigenic effects by reducing expression of dual specificity phosphatase 4 (DUSP4), which negatively regulates ERK and p38 activity [46]. These reports collectively highlight the significance of AKT and ERK in cell proliferation. In our study, concurrent treatment with IL-4/IL-13 and insulin leads to increased ERK phosphorylation. This might be ascribed to upregulated IRS-1 tyrosine phosphorylation (Figure 4C) and/or IL-13Rα2 expression (RNA-seq results; Table S1).

IL-4 and IL-13 stimulates tyrosine phosphorylation of STAT6 and IRS [5]. Here we also found that prolonged treatment with IL-4 and IL-13 augmented IRS-1 expression via STAT6. This is consistent with observations that STAT6 is highly activated and IRS-1 protein is much more abundant in mammary glands during pregnancy, when there is a Th2-dominant cytokine milieu [13,47,48]. In normal human breast tissues and ductal carcinoma in situ, expression patterns of IRS-1 correlate with those of STAT6 and p-STAT6 [49]. This supports our finding that IL-4 and IL-13 stimulate IRS-1 expression via STAT6. Upregulation of IRS-1 by IL-4 has also been observed in osteoblasts, in which sustained exposure to IL-4 enhances IRS-1 expression and restores insulin sensitivity [50]. Similarly, prolonged treatment with IL-13 results in increased IRS-2 expression and AKT activity in beta-cells, which might mediate the pro-survival effect of IL-13 [51].

Regulation of IRS expression occurs at the transcriptional and post-transcriptional levels. Here we discovered that IL-4 and IL-13 enhanced IRS-1 protein abundance via STAT6. Increased expression of IRS-1 mRNA was also observed (Table S1). STAT6 is a transcription factor; however, no STAT6-binding sites were identified in the promoter of the *Irs1* gene. Instead, there are AP-1, Sp1 sites and estrogen response element (ERE) half sites [52]. There is evidence that STAT6 regulates gene expression through interacting with Sp1 [53]. Whether similar mechanism contributes to transcription of the *Irs1* gene merits further investigation. Several microRNAs and E3 ubiquitin ligases control IRS expression at post-transcriptional level [54,55]. STAT6 might target these genes, which in turn, govern IRS-1 mRNA degradation, translation, or protein degradation.

IRS-1 is the key molecule for insulin, IGF and IL-4/IL-13 signaling. In mammary cells, upregulation of IRS-1 by IL-4 and IL-13 amplifies the initially weak activation of IRS-1 (Figure 4B). It also exerts a priming effect to augment insulin signaling (Figure 5). Accumulating evidence has demonstrated the beneficial effect of Th2 cytokines on diabetes mellitus (DM) and related metabolic diseases. In a type II DM mouse model, IL-4 improved insulin sensitivity and glucose tolerance [56]. Studies of *Il-13* gene ablation have revealed that IL-13 controls hepatic glucose production by downregulating enzymes involved in gluconeogenesis [57]. It is not clear if IL-4 and IL-13 improve insulin sensitivity by increasing IRS expression in other tissues, but they do so in osteoblasts and beta-cells [50,51].

Cellular responses are controlled by integration of external stimuli in the microenvironment. In mammary cells, crosstalk between IL-4/IL-13 and insulin signaling confer optimal cell proliferation (Figure 8). Similar signaling crosstalk has been documented. Regarding DM, IL-4 potentiates insulin signaling, leading to enhanced AKT activity [56]. Hence, Th2 immune skewing such as during chronic helminth infections improves insulin sensitivity [58]. In the tumor microenvironment, Th2 cytokines act on tumor cells or stromal cells. Regarding the latter, IL-4 and IL-13 stimulate differentiation of M2 macrophages in tumor stroma, which favors tumor progression [59,60]. In a mouse medulloblastoma model, tumor-derived astrocytes secrete IL-4 to stimulate microglia for the production of IGF-I, which, in turn, promotes tumor growth [61]. Likewise, M2-like tumor-associated macrophages polarized by IL-4/IL-13 promote stemness and metastasis of thyroid cancer cells by secreting IGF-I and IGF-II [62]. Under these conditions, a sequential action of IL-4/IL-13 and IGF-I targeting on macrophages and cancer cells, respectively, culminates in cancer progression.

In conclusion, IL-4 and IL-13 are pleiotropic cytokines. They exert functions beyond host defense and immune regulation. During pregnancy, IL-4 and IL-13 facilitate mammary gland development by promoting cell proliferation via STAT6 and IRS-1.

## 4. Materials and Methods

### 4.1. Reagents

Recombinant murine IL-4 and IL-13 were purchased from PeproTech (Rocky Hill, NJ, USA). Antibodies to β-casein (Cat# sc-17971), STAT6 (Cat# sc-981) and ERK1 (Cat# sc-93) were obtained from Santa Cruz Biotechnology (Santa Cruz, CA, USA). Antibodies to phosphotyrosine (clone 4G10; Cat# 05-321) and IRS-1 (Cat# 06-248) were supplied by Millipore (Temecula, CA, USA). Antibody to phospho-STAT6 (Y641) (Cat# ab54461 and Cat# ab263947) was obtained from Abcam (Cambridge, UK). Antibodies to phospho-AKT (S473) (Cat# 9271), AKT (Cat# 9272) and phospho-ERK (T202/Y204) (Cat# 9101) were obtained from Cell Signaling (Beverly, MA, USA). Antibody to PCNA (Cat# 610664) was obtained from BD Biosciences (San Jose, CA, USA). Antibody to actin (Cat# A-5060) was obtained from Sigma-Aldrich (St. Louis, MO, USA). STAT6 inhibitor AS1517499 was purchased from Axon Medchem (Groningen, The Netherlands). IRS inhibitor NT157 was obtained from MedChem Express (Monmouth Junction, NJ, USA).

### 4.2. Cell Culture

All experiments were conducted with first or second passage mammary epithelial cells as described [63]. Mammary alveoli were isolated from mid-pregnant ICR mice and plated onto Matrigel (Corning, Bedford, MA, USA) in nutrient mixture F-12 (Sigma-Aldrich, St. Louis, MO, USA) containing 10% fetal bovine serum (Gibco, Carlsbad, CA, USA), 1 mg/mL fetuin (Sigma-Aldrich), 5 ng/mL EGF (Sigma-Aldrich), 5 μg/mL insulin (Sigma-Aldrich), and 1 μg/mL hydrocortisone (Sigma-Aldrich). After 72 h, cells were cultured in Dulbecco’s modified Eagle’s medium (DMEM)/nutrient mixture F-12 (Gibco) containing hydrocortisone and insulin and stimulated with IL-4 or IL-13 (50 ng/mL). Alternatively, cells were trypsinized and cultured onto Matrigel or tissue culture plastic overnight. The culture medium was then changed to DMEM/F-12 containing hydrocortisone and insulin and cells were stimulated with cytokines. For insulin signaling experiments (Figure 4A,B and Figure 5), insulin was omitted from the cultures [63]. In this study, animals were obtained, maintained, and used in accordance with the policies of the Institutional Animal Care and Use Committee of the Chung Shan Medical University (IACUC Approval No 1390, 2070 and 2155).

### 4.3. RNA Interference

Lentivirus containing small hairpin RNA (shRNA) against *Irs1* (targeting sequence: 5′-GACGCTCCAGTGAGGATTTAA-3′; TRCN0000244225) and pseudotyped lentivirus scramble control (TRC2.Scramble) were obtained from the National RNAi Core Facility (Academia Sinica, Taipei, Taiwan). Second passage mammary epithelial cells were plated onto tissue culture plastic overnight and infected with lentivirus. After 48 h, cells were cultured in DMEM/F-12 medium containing hydrocortisone and insulin and then stimulated with IL-4 or IL-13 for 2 d.

### 4.4. Immunoprecipitation and Western Blot Analysis

Cells were lysed in lysis buffer containing 50 mM Tris (pH 7.4), 150 mM NaCl, 2 mM EDTA, 1 mM Na_3_VO_4_, 10 mM NaF, 10 μg/mL aprotinin, 10 μg/mL leupeptin, 1 mM phenylmethylsulfonyl fluoride, and 1% Triton X-100. Whole cell lysates were incubated with 1–2 µg of antibody and 30–50 µL of protein A-Sepharose beads (Invitrogen, Rockford, IL, USA) at 4 °C for 4–6 h. Immunoprecipitates or whole cell lysates were resolved by SDS-PAGE, transferred to polyvinylidene fluoride (PVDF) membranes, and probed with antibodies to β-casein (0.4 μg/mL), actin (1:1000), PCNA (0.25 μg/mL), phospho-STAT6 (1:1000), STAT6 (0.4 μg/mL), phosphotyrosine (4G10; 1 μg/mL), IRS-1 (1 μg/mL), phospho-AKT (1:500), phospho-ERK (1:500), AKT(1:500), and ERK (0.4 μg/mL). Signals were detected by enhanced chemiluminescence reagents (Millipore, Billerica, MA, USA). Actin was used as a loading control. ImageJ (NIH, Bethesda, MD, USA) was used to quantify band intensity.

### 4.5. Real-Time PCR

Total RNA was isolated with TRIzol (Invitrogen, Waltham, MA, USA), and treated with DNase I (Promega, Madison, WI, USA). The first strand cDNA synthesis was carried out with SuperScript III kit (Invitrogen). All real-time PCR reactions were conducted with an ABI Prism-7000 system using the Power SYBR Green PCR Master Mix (Life Technologies, Carlsbad, CA, USA). The PCR primers used for mouse *Il4ra* were 5′-CCTCTGCATCCCGTTGTT-3′ (forward) and 5′-CTTGGTTGACTCCTGGCTTC-3′ (reverse); mouse *Il13ra1* were 5′-TGATGACCAACAGGATAAGAAAAT-3′ (forward) and 5′-CAGCGGACTCAGGATCAC-3′ (reverse); mouse *Ym1* were 5′-TGAATGAAGGAGCCACTGAG-3′ (forward) and 5′-AAAGTAGATGTCAGAGGGAAATGTC-3′ (reverse); mouse *18S ribosomal RNA* (*rRNA*) were 5′-GTAACCCGTTGAACCCCATT-3′ (forward) and 5′-CCAT CCAATCGGTAGTAGGG-3′ (reverse). *18S rRNA* was used as an internal control. The ratio of each target mRNA to *18S rRNA* (fold change) was calculated as 2^−∆Ct^, and ΔCt = Ct_target gene_ − Ct_18s rRNA_.

### 4.6. Cell Proliferation Assay

Second passage mammary epithelial cells cultured on Matrigel (growth factor-reduced; Corning, Bedford, MA, USA)-coated or uncoated coverslips were stimulated with IL-4 or IL-13 for 1 or 2 d. Before harvesting, cells were pulsed with 10 μM EdU for 8 or 15 h. Cells cultured on Matrigel were fixed in methanol:acetone (1:1), and cells cultured on uncoated coverslips were fixed in 4% paraformaldehyde and permeabilized by 0.5% Triton X-100. EdU incorporation was detected using Click-iT^TM^ Plus EdU Alexa Fluor^TM^ 488 Imaging Kit (Cat# 10637, Life Technologies, Carlsbad, CA, USA). For cells cultured on uncoated coverslips, the percentage of EdU-positive cells was determined by TissueFAXS^TM^ cytometry (TissueGnostics, Vienna, Austria).

### 4.7. Immunofluorescence Microscopy

Second passage mammary epithelial cells cultured on Matrigel (growth factor-reduced) were fixed in cold methanol:acetone (1:1) at −20 °C overnight, blocked with 1% goat serum for 30 min, and incubated with rhodamine phalloidin (5 U/mL; Life Technologies, Carlsbad, CA, USA) at room temperature for 20 min, followed by two washes with PBS. Nuclei were stained with Hoechst 33342 (5 μg/mL; Life Technologies) at room temperature for 10 min and washed twice with PBS. After mounting the samples on glass slides, cells were observed under a confocal laser scanning microscope (Zeiss LSM 510 META).

### 4.8. Statistical Analysis

Data are expressed as mean ± S.E.M. of at least three independent experiments, and were analyzed by one-way analysis of variance (ANOVA) followed by Tukey’s multiple-comparisons test. *p* < 0.05 was considered statistically significant. All statistical analyses were performed using GraphPad Prism 5 software (GraphPad Software, San Diego, CA, USA).

## Data Availability

The data presented in this study are available on request from the corresponding author.

## Supplementary Materials

The following are available online at https://www.mdpi.com/article/10.3390/ijms222112008/s1.
