# Supplementary material for: IL-4 and IL-13 Promote Proliferation of Mammary Epithelial Cells through STAT6 and IRS-1
Source: Int J Mol Sci. 2021 Nov 5;22(21):12008. doi: 10.3390/ijms222112008 (PMC8584551; doi:10.3390/ijms222112008)
Supplement: Supplementary file 1 [file ijms-22-12008-s001.zip › ijms-1208714-supplementary.pdf]

Supplementary Materials

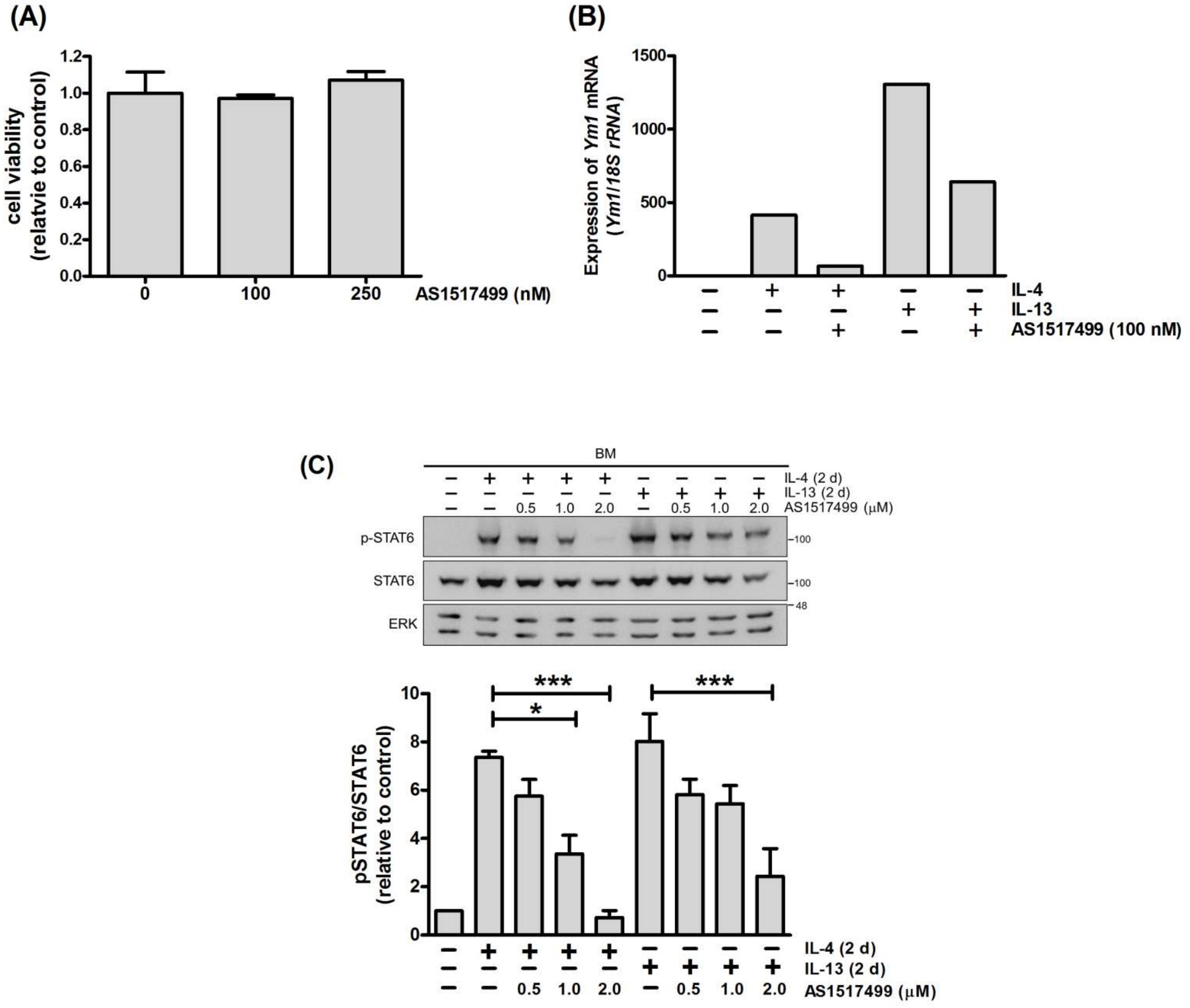

Figure S1. Effect of STAT6 inhibitor AS1517499 on cell viability, *Ym1* mRNA expression and STAT6 phosphorylation. **(A)** Mammary cells cultured on basement membrane (BM)-like matrix, Matrigel, were treated with AS1517499 (100, 250 nM) for 1 d. Cells were harvested and viable cells were counted. Data were expressed as fold change relative to control. (**B**) Cells were pretreated with AS1517499 (100 nM) for 1 h and stimulated with IL-4 or IL-13 for 16 h. Total RNA was subjected to real time RT-PCR with primers for *Ym1* and *18S rRNA*. *18S rRNA* was used as an internal control. Expression of *Ym1* mRNA were normalized to internal control and expressed as fold change relative to control sample. (**C**) Cells were pretreated with STAT6 inhibitor AS1517499 (0.5~2 μM) for 1 h and stimulated with IL-4 or IL-13 for 2 d. Total cell lysates were analyzed by immunoblotting with antibodies to phospho-STAT6 (p-STAT6), STAT6 and ERK. ERK was used as a loading control. Data were quantified, normalized to loading control and expressed as fold change (pSTAT6/STAT6) relative to control. * $p < 0.05$, *** $p < 0.005$.

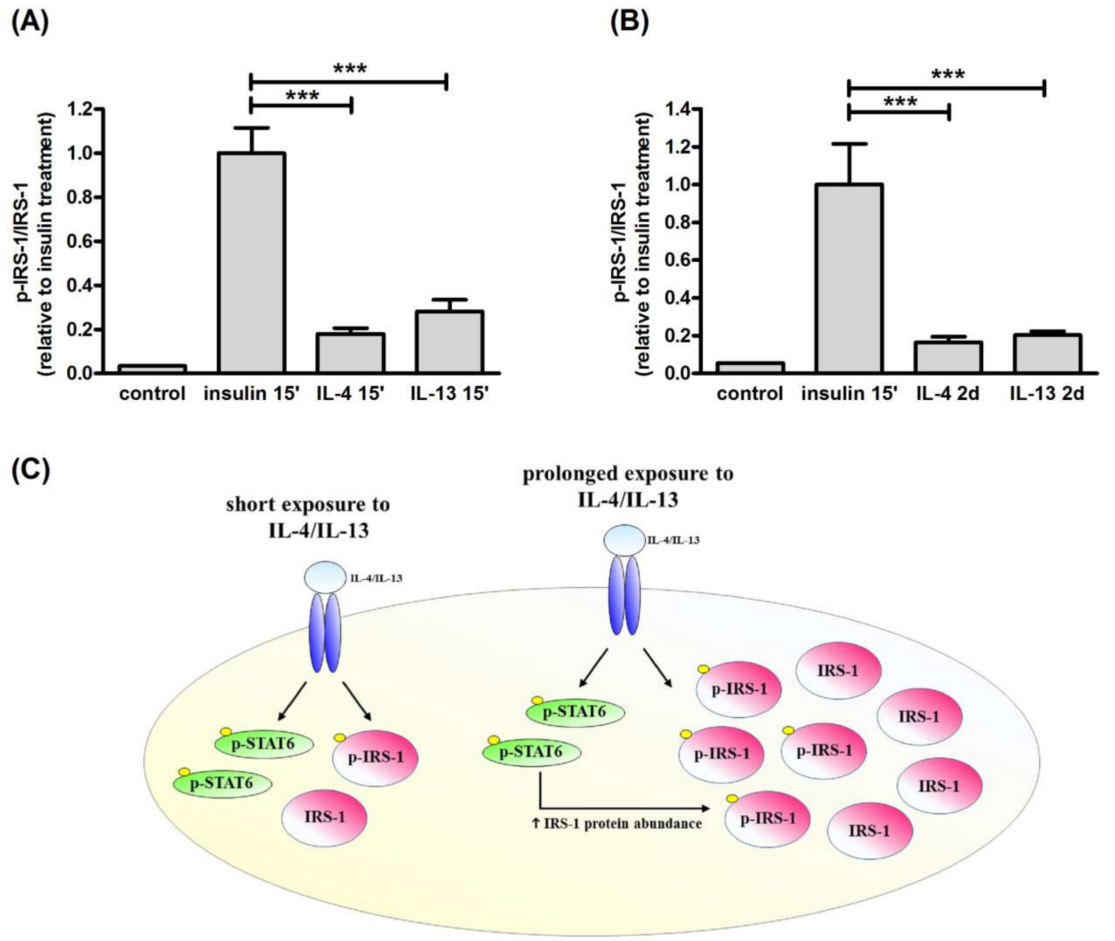

Figure S2. The ratio of IRS-1 tyrosine phosphorylation to IRS-1 expression (p-IRS-1/IRS-1) in figures 4A and 4B. (**A, B**) The ratios of IRS-1 tyrosine phosphorylation to IRS-1 expression (p-IRS-1/IRS-1) in figures 4A and 4B were calculated and expressed as fold change relative to insulin treatment. *** $p < 0.005$. (**C**) Summary diagram for results in A and B. Short exposure to IL-4/IL-13 leads to low levels of IRS-1 tyrosine phosphorylation. Prolonged cytokine treatment causes an increase in levels of IRS-1 tyrosine phosphorylation, due to enhanced IRS-1 protein abundance. In other words, prolonged treatment does not upregulate JAK activity, but substantially increases amounts of JAK substrate, IRS-1, to be phosphorylated. Yellow circles in the diagram indicate cytokine-stimulated tyrosine phosphorylation.

Table S1. RNA-sequencing results. Mammary cells cultured on Matrigel were untreated or treated with IL-4 or IL-13 for 12 h. Total RNA was subjected RNA-seq analysis.

	FPKM		
	**Control**	**IL-4**	**IL-13**
Il4ra	18.61	25.91	27.30
Il13ra1	18.96	18.84	18.98
Il13ra2	0.16	5.64	15.01
Irs1	12.82	31.17	29.76
